# Clinical, molecular, and histological characteristics of severely necrotic and fatal mpox in HIV-infected patients

**DOI:** 10.1186/s12981-023-00580-6

**Published:** 2023-11-27

**Authors:** Sandra Rajme-López, Ever A. Corral-Herrera, Andrea C. Tello-Mercado, Karen M. Tepo-Ponce, Raúl E. Pérez-Meléndez, Ángela Rosales-Sotomayor, Grecia Figueroa-Ramos, Karla López-López, Judith G. Domínguez-Cherit, Oswaldo San-Martín-Morante, Marcela Saeb-Lima, Armando Gamboa-Domínguez, Alfredo Ponce-de-León, Brenda Crabtree-Ramírez, Pilar Ramos-Cervantes, Guillermo M. Ruíz-Palacios

**Affiliations:** 1 Infectious Diseases Department, Instituto Nacional de Ciencias Médicas y Nutrición, “Salvador Zubirán” Vasco de Quiroga #15, Belisario Domínguez Sección XVI, Tlalpan, Ciudad de México, 14080 México; 2 Internal Medicine Department, Instituto Nacional de Ciencias Médicas y Nutrición “Salvador Zubirán”, Mexico City, Mexico; 3 Dermatology Department, Instituto Nacional de Ciencias Médicas y Nutrición “Salvador Zubirán”, Mexico City, Mexico; 4 Pathology Department, Instituto Nacional de Ciencias Médicas y Nutrición “Salvador Zubirán”, Mexico City, Mexico; 5 Virology and Molecular Biology Laboratory, Instituto Nacional de Ciencias Médicas y Nutrición “Salvador Zubirán”, Mexico City, Mexico

**Keywords:** Mpox, Severe Disease, HIV, Histopathology

## Abstract

**Background:**

This case series of 5 patients with severely necrotic mpox highlights the predominantly necrotic nature of lesions seen in cases of severe mpox as shown by skin and lung biopsy, as well as the extensive dissemination of the infection, as shown by polymerase chain reaction (PCR) assessment in different body sites.

**Case presentations:**

Patients were male, the median age was 37, all lived with HIV (2 previously undiagnosed), the median CD4^+^ cell count was 106 cells/mm^3^, and 2/5 were not receiving antiretroviral treatment. The most common complication was soft tissue infection. Skin and lung biopsies showed extensive areas of necrosis. Mpox PCR was positive in various sites, including skin, urine, serum, and cerebrospinal fluid. The initiation of antiretroviral treatment, worsened the disease, like that seen in immune reconstitution syndrome. Three patients died due to multiple organ failure, presumably associated with mpox since coinfections and opportunistic pathogens were ruled out.

**Conclusions:**

Severely necrotic manifestations of mpox in people living with advanced and untreated HIV are related to adverse outcomes.

## Background

Monkeypox virus is currently the most important cause of Orthopoxvirus infection in humans [1]. Death appears to be infrequent in immunocompetent hosts [2–5], but there seems to be a correlation between the host´s immunological status and the severity of the disease [4, 6–8]. The case-fatality rate of mpox reported in patients with HIV infection is up to 10% [9–12]. This report aims to describe the clinical course of patients with extensive and severely necrotic mpox, emphasizing histopathological and virological characteristics.

## Case presentations

### Case 1

Thirty-year-old male without previous known medical history. He began on August 2022 with a single skin lesion in the glans penis. During the following days, similar lesions appeared throughout the trunk, neck, face, scalp, arms, and legs. After medical evaluation, he was started on valacyclovir, without clinical improvement. Positive skin PCR confirmed mpox. Two weeks later he developed facial edema, sialorrhea, poor oral intake, and worsening of the skin lesions, with phimosis and skin necrosis of the genital area.

On September 2022 he was referred to our Institution. On admission, he was afebrile, tachycardic (140 bpm), tachypneic (18 rpm), and hypoxemic (SpO2 89% on room air). Physical examination revealed a disseminated polymorphic dermatosis affecting all body segments (> 200 lesions) composed of giant ulcerative and crusted lesions with an edematous and indurated border surrounded by erythema; early lesions were pustules and umbilicated papules. HIV serology was positive (121 CD4^+^ cells/mm^3^ – 5% / viral load 312,077 copies/mL). HSV-1 coinfection was confirmed by PCR. Other sexually transmitted diseases were excluded.

Tracheal stricture and 2 pulmonary nodules were identified on admission computed tomography (CT) scan. Magnetic resonance imaging (MRI) of the pelvis showed signs of bilateral orchitis, urethritis, and superficial necrotic tissue around the penis body and glans. An emergency tracheostomy was performed to ensure airway patency. He was started on meropenem, vancomycin, acyclovir, and prophylactic trimethoprim-sulfamethoxazole. Six days later, after excluding opportunistic infections, antiretroviral therapy was initiated (bictegravir/tenofovir/emtricitabine).

Two weeks later, the patient´s clinical condition continued to deteriorate. Due to the unavailability of poxvirus antiviral agents, the local investigation board review (IRB) authorized the infusion of plasma from a previously healthy volunteer who had received two doses of the JYNNEOS vaccine > 28 days prior (the patient gave explicit consent for this extraordinary therapeutic measure). He received the infusion without complications but continued worsening thereafter (progression of skin lesions, preseptal cellulitis, increasing facial edema, acute kidney injury, neurologic deterioration, and respiratory failure). Subsequent chest CT revealed an increase in the size and number of pulmonary nodules. He was started on empiric liposomal amphotericin B, but he developed refractory shock and multiple organ failure (coagulopathy, elevated liver enzymes). He ultimately died four weeks after his admission. An extensive microbiological evaluation was negative for other pathogens. Serum PCR stayed positive for 28 days.

### Case 2

Forty-four-year-old male with a past medical history of type 2 diabetes mellitus, with poor adherence to lifestyle changes and pharmacological treatment. He began on September 2022 with fever, malaise, myalgia, arthralgia, and disseminated rash characterized by vesicles, pustules, crusts, and eschar, as well as necrosis of the 5th left toe. Mpox was confirmed by PCR testing of skin lesions; he received symptomatic treatment.

On October 2022 he was referred to our Institution due to clinical worsening of the skin lesions. On admission, he was afebrile, tachycardic (110 bpm), and tachypneic (22 rpm), with normal oxygen saturation. Physical examination revealed a diffuse skin rash (> 100 lesions), with characteristic vesicular, pustular, umbilicated, and crusted lesions. The perianal region was notable for 2 ulcers with irregular borders. HIV serology was positive (106 CD4^+^ cells/mm^3^ – 6% / viral load 726,454 copies/mL). HSV-2 coinfection was confirmed by PCR. Other sexually transmitted diseases were excluded.

Multiple pulmonary nodules were identified by chest CT scan. An abdominopelvic CT scan revealed concentric thickening of the rectum with intramural collections, a perianal abscess, and a trans-sphincteric anal fistula. The patient was started on piperacillin-tazobactam, vancomycin, acyclovir, fluconazole, and prophylactic trimethoprim-sulfamethoxazole. Complete surgical drainage of the perianal abscess was performed, without complications. Six days after starting fluconazole, he presented with dysphagia. Upper endoscopy revealed elevated lesions with central umbilication in the distal esophagus, similar to those seen on the skin. No local treatment was applied, and he continued on fluconazole. Lung biopsy excluded infectious/malignant causes of the pulmonary nodules; mpox virus PCR was positive. After the exclusion of opportunistic infections, antiretroviral therapy was initiated (bictegravir/tenofovir/emtricitabine).

The patient´s skin lesions progressed to necrotic ulcerations in the following days. He developed acute kidney injury, respiratory failure, and distributive shock. Due to neurologic impairment, a lumbar puncture was performed. Cerebrospinal fluid (CSF) analysis was non-specific (WBC 0 mm3, RBC 465 cells/mL, protein 36.9 mg/dL, glucose 99 mg/dL); mpox virus PCR was positive. Despite orotracheal intubation, vasopressors, and renal replacement therapy, he developed multiple organ failure (coagulopathy, elevated liver enzymes, cardiac dysfunction) and died. An extensive microbiological evaluation was negative for other pathogens. Serum PCR stayed positive for 30 days.

### Case 3

Thirty-seven-year-old male with recently diagnosed HIV infection (25 CD4^+^ cells/mm^3^ – 3% / viral load 31,385 copies/mL), and on < 1 month of antiretroviral treatment (bictegravir/tenofovir/emtricitabine). His past medical history was also positive for *T. pallidum* and *C. trachomatis* infections (previously treated). He began on September 2022, with localized dermatosis affecting the face, characterized by umbilicated papules.

On October 2022 he was referred to our Institution for dissemination of the dermatosis. On admission, he was afebrile, tachycardic (107 bpm), and without respiratory abnormalities. Physical examination revealed a disseminated polymorphic dermatosis (< 50 lesions) affecting all body segments, with central ulceration and crusting, indurated border, and surrounded by erythema. Oral candidiasis and necrotic proctitis were identified. The right hand and forearm were erythematous, with edema and disproportionate pain. CT imaging revealed cellulitis of the right distal forearm and hand. HSV-2 coinfection was confirmed by PCR. Other sexually transmitted diseases were excluded. The patient was started on piperacillin-tazobactam, vancomycin, fluconazole, doxycycline, acyclovir, and prophylactic trimethoprim-sulfamethoxazole.

He underwent anal canal surgical debridement and fasciotomy of the right forearm due to compartment syndrome. Notably, characteristic lesions were documented at surgical incision sites, which rapidly enlarged over the next days. Days later, he developed progressing respiratory distress. On head and neck CT, diffuse inflammation was observed in the buccopharyngeal space, with segmental obliteration of the glottis. He underwent emergency surgical tracheostomy, but complete occlusion of the airway conditioned severe hypoxemia and cardiac arrest. He died 21 days after admission. No opportunistic infections were identified. Serum PCR stayed positive for 21 days.

### Case 4

Twenty-five-year-old male with HIV infection, on 6 months of antiretroviral treatment (68 CD4^+^ cells/mm^3^ – 7% / viral load 367 copies/mL). He was referred to our Institution on October 2022 for progressive mpox. Physical examination showed 2–3 cm round verrucous lesions with yellow crust, affecting the scalp, neck, torso, forearms, penis, and right foot. Soft tissue infection by methicillin-sensitive *S. aureus* was diagnosed. After a 2 week course of antibiotic therapy, he was discharged. Two months later he had no mpox lesions.

### Case 5

Fifty-eight-year-old male with HIV infection, on > 6 months of antiretroviral treatment (177 CD4^+^ cells/mm^3^ – 8% / suppressed viral load). He was referred to our Institution on October 2022 to evaluate a large necrotic ulcer in the perianal region. Physical examination revealed a large ulcer (10 × 6 cm) in the perianal area with secondary bacterial infection, plus some disseminated crusted ulcers and umbilicated papules and vesicles, with necrotic center. Broad-spectrum antibiotics were started, and extensive surgical debridement was performed. Tissue cultures were positive for *E. coli, E. faecium*, and *A. haemolyticum*. After ten days of antibiotic therapy, he was discharged. Three months later, he still had mpox lesions (crusts) on the thighs, glutes, and perianal region. Virological assessment at that point was not made. Table 1 presents a detailed comparison of the clinical and molecular characteristics of the 5 cases described.

**Table 1:** Clinical and molecular characteristics of severely necrotic mpox in HIV-infected patients

	Age / gender	Smallpox vaccination history	CD4^+^ count (cells/mm^3^) / HIV RNA (copies/mL) / HAART	Other comorbidities	Coinfections at time of mpox diagnosis	Molecular diagnosis, PCR (ct value)	Skin lesions (number)	CNS	Lung	Eye	Lymphadenopathy	Other organs	Complications	Mpox treatment	Outcome
Case 1	30/ Cis-gender man	No	121 (5%) / 312,077 / Untreated	No	HSV-1	Skin: positive (22) Throat: positive (26) Serum positive (23) Plasma: positive (21) Urine: positive (24)	> 200 (all body segments)	No	Probable (nodules on CT)	No	Yes (cervical, axillary, mediastinal)	Multiple organ failure	Preseptal cellulitis Tracheal stricture Orchitis Phimosis	Vaccinated donor plasma	Death
Case 2	44/ Cis-gender man	No	106 (6%) / 726,454 / Untreated	Type 2 Diabetes	HSV-2	Skin: positive (NA) Throat: positive (24) Serum positive (29) Plasma: positive (29) Urine: positive (28) Esophagus: positive (24) Lung: positive (23) CSF: positive (35)	100–200 (all body segments)	Yes (MPXV PCR + on CSF)	Yes (nodules on CT, MPXV PCR + on biopsy)	No	No	Multiple organ failure	Perianal abscess/fistula Esophagitis	No	Death
Case 3	37/ Cis-gender man	No	25 (3%) / 31,385 / Recent start	No	HSV-2 Oral candidiasis	Skin: positive (NA) Throat: positive (25) Serum positive (25) Plasma: positive (24) Urine: positive (35)	< 50 (all body segments)	No	No	No	Yes (axillary, inguinal)	Multiple organ failure	Cellulitis / compartmental syndrome Proctitis Tracheal stricture	No	Death
Case 4	25/ Cis-gender man	No	68 (7%) / 367 / 6 month after initiation	No	No	Skin: positive (NA)	50–100 (all body segments)	No	Possible (micro-nodules on CT)	No	No	Hepatomegaly	Impetiginization Cellulitis	No	Alive
Case 5	58/ Cis-gender man	No	177 (8%) / Undetectable/ Irregular adherence	No	HSV-2 HPV	Skin: positive (NA)	50–100 (all body segments)	No	No	No	No	No	Perianal abscess	No	Alive

Abbreviations: CMV (cytomegalovirus), CNS (central nervous system), ct (cycle threshold), CT (computed tomography), HAART (highly active antiretroviral therapy), HPV (human papillomavirus), HSV (herpes simplex virus), IV (intravenous), MAC (*Mycobacterium avium* complex) MPXV (mpox virus), NA (not available), PCR (polymerase chain reaction)

## Histopathological findings

**Fig. 1:**
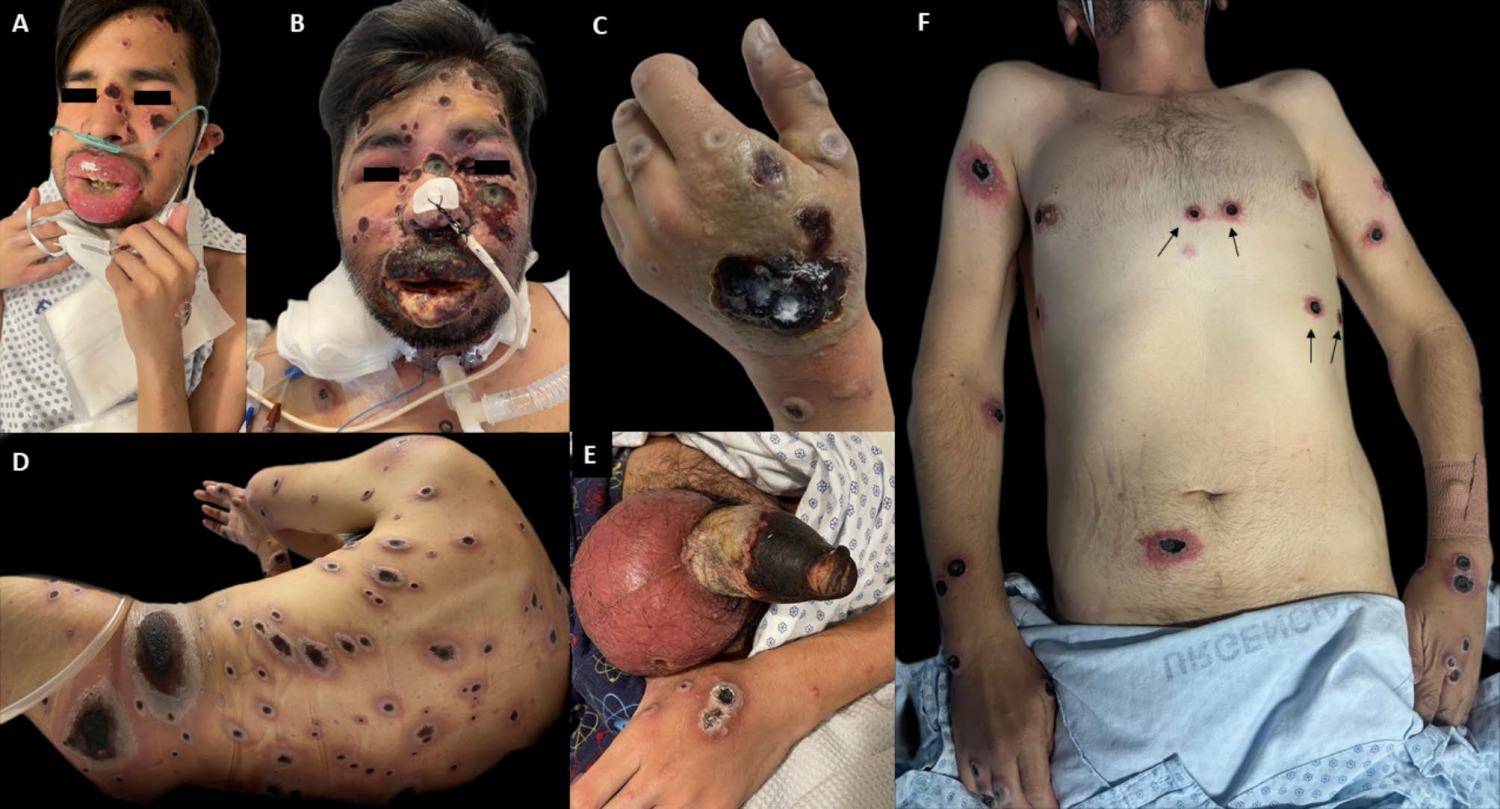
Clinical images of patients with severely necrotic mpox. **A** and **B**. Mucosal edema with progression to extensive necrosis. **C** and **D**. Giant skin ulceration. **E**. Phimosis and penile necrosis. **F**. Pseudo-Koebner phenomenon (mpox lesions appeared on EKG monitoring sites)

**Fig. 2:**
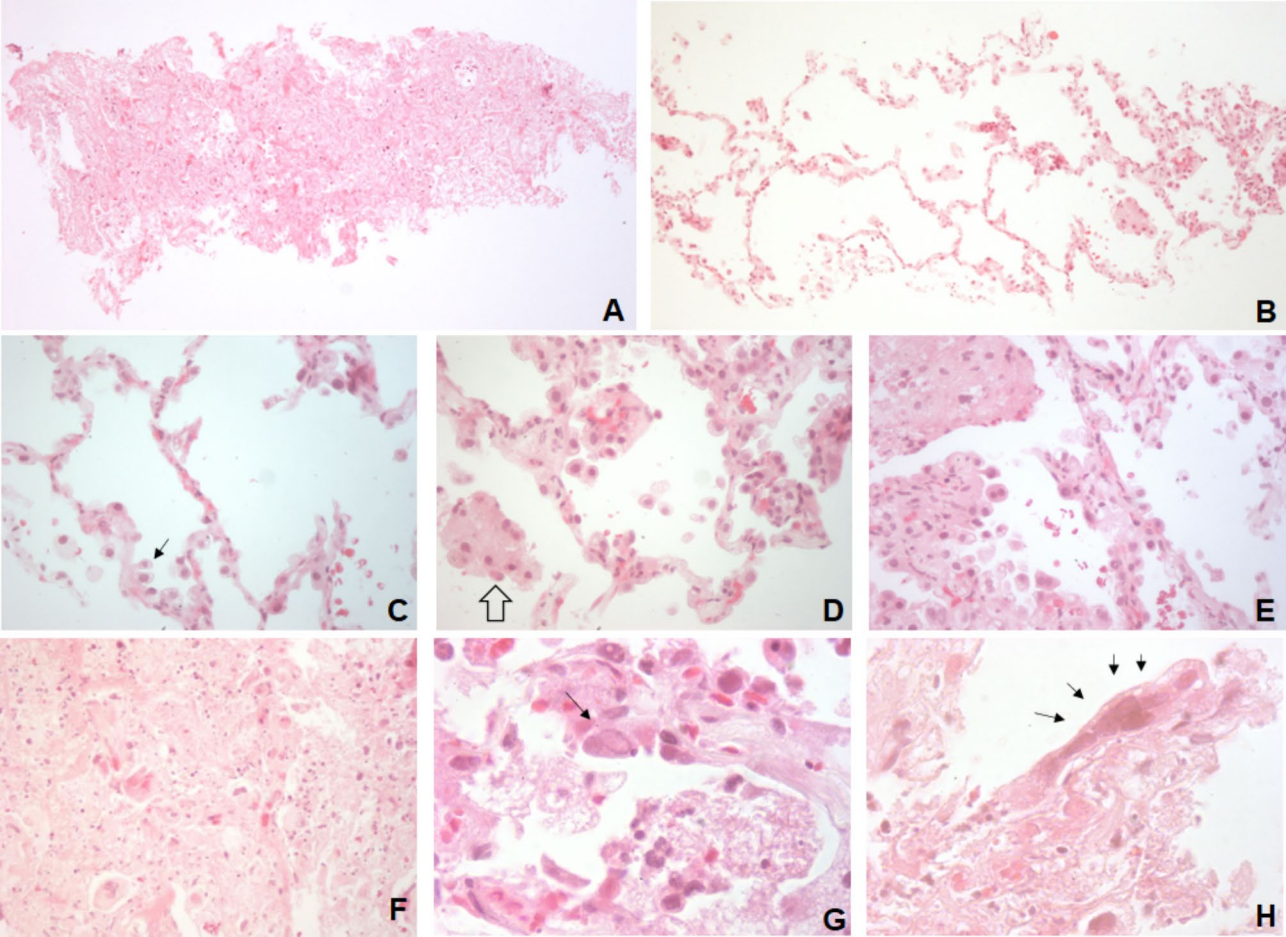
Lung tissue with broncho-pneumonic changes. Congestive septi (**A**) and effacement of alveolar pattern with necrosis (**B**) Both 40X. Prominent type II pneumocytes (arrow) and congestive septi (**C**), followed by desquamative changes (wide arrow) and interstitial inflammation and macrophages in alveolar space (**D**). Necrotic debris in alveoli with few erythrocytes and wide congestive septum (**E**). Parenchymal necrosis with karyorrhexis and giant histiocytic cells (**F**). All sequential alveolar changes at 400X. Cytopathic changes in pneumocytes (arrows). Ground glass chromatin in a large cell lining alveoli filled with fibrin, polymorphonuclear cells, macrophages, and lymphocytes (**G**). Sincitial cell covering alveolar surface close to necrotic area (**H**). Both Fig. 1000X.

Clinical dermatological findings are displayed on Fig. 1. Skin biopsies (Fig. 2) showed a partially necrotic epithelium with scale, crust, and mounds of parakeratosis. Marked acanthosis, vacuolated keratinocytes with bluish-gray cytoplasm and enforcement of nucleus membrane, and some multinucleated giant cells were observed as distinctive of Mpox infection. A lymphoplasmacytic inflammatory infiltrate in the superficial mucosa accompanied these changes. Periodic acid Schiff (PAS), Ziel-Nielsen (ZN), Fite Faraco, and Gram stains were negative.

**Fig. 3:**
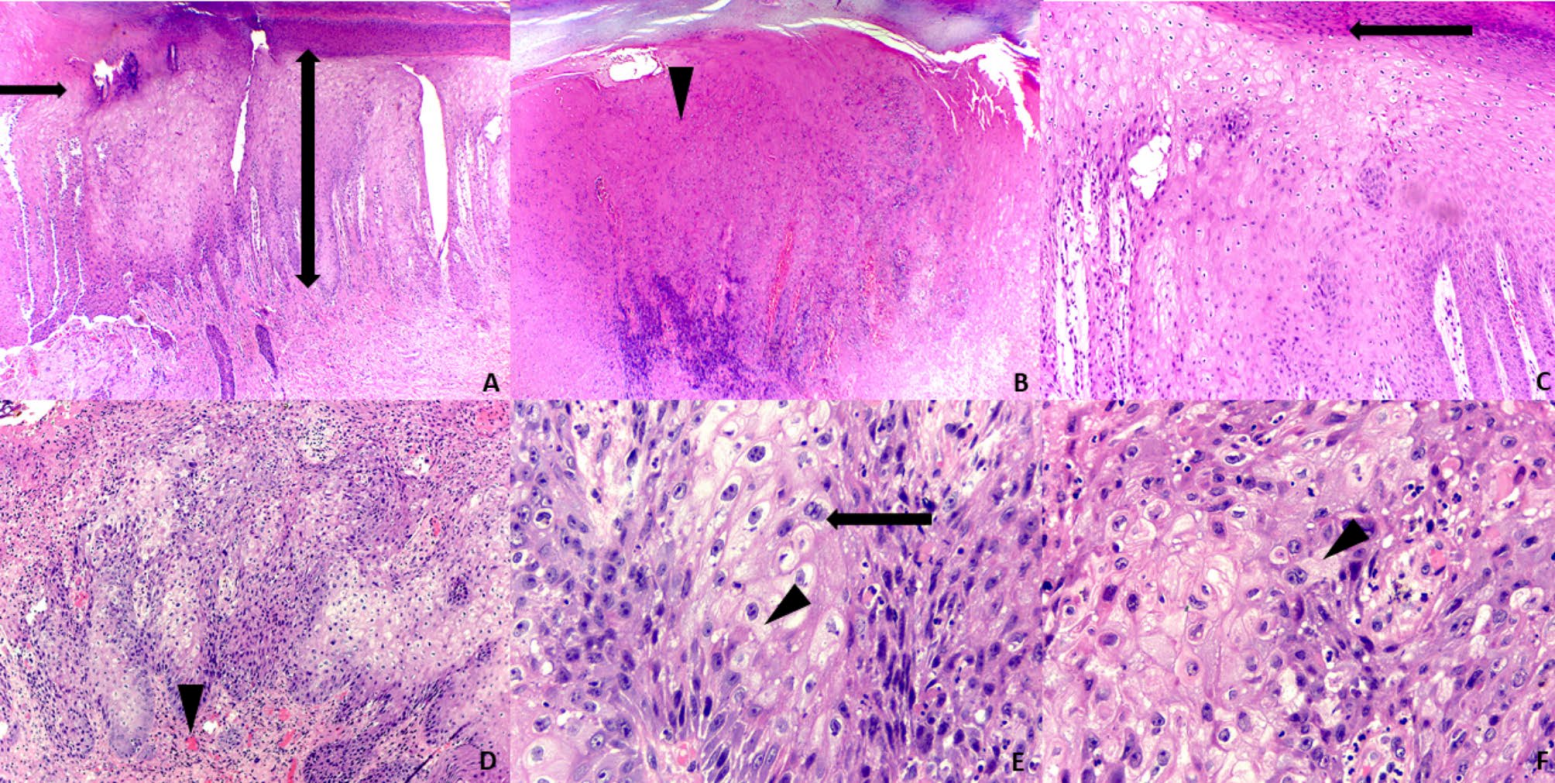
Skin biopsies. (**A**) Partially necrotic epithelium (arrow) and marked acantosis (two-headed arrow). (**B**) Scale crust (arrow head). (**C**) Mounds of parakeratosis (arrow). (**D**) Lymphoplasmacytic inflammatory infiltrate (arrow head), (**E**) Multinucleated keratinocytes (arrow). **E** and (**F**) Vacuolated keratinocytes (arrow heads). (Hematoxylin and eosin)

Lung biopsy (Fig. 3) displayed areas of necrosis and foci of irregularly shaped cells with nucleomegaly, accompanied by a predominantly lymphocytic infiltrate. On high-power fields, syncitia formation with multinucleated pneumocytes were observed. Ground glass chromatin was identified, suggesting cytopathic damage. Immunohistochemistry reactions for citomegalovirus, adenovirus, and herpes simplex virus were negative.

## Discussion and conclusions

This severely necrotic form of mpox was more frequent than previously reported [2, 5], accounting for 6% of all cases of mpox seen at our institution during the time these cases presented (5/81). Of note, from 7 hospitalized mpox patients at our center, these cases were the only ones with this severely necrotic form of the disease and were the only ones with CD4^+^ cell count < 200 cell/mm^3^.

All cases displayed features resembling disseminated smallpox. Cases 1, 2, and 3 experienced a clinical syndrome similar to smallpox of the hemorrhagic variety [13], with thrombocytopenia, prolongation of thrombin and prothromboplastin times, and gastrointestinal bleeding. The hemorrhagic types of smallpox were the deadliest ones, with an estimated case fatality rate of 60–90%, even in vaccinated people [13], contrasting with the observations of a less clinically severe disease after mpox infection or vaccination [14].

Complications from severe smallpox occurred at a later stage of the disease. Typically, patients remained febrile for various days, after which pulmonary or neurological manifestations began to appear. The cases shown here presented a similar chronology of events, probably reflecting the physiopathological similarities between smallpox and mpox.

The neurological manifestations described in case 2, in whom the PCR in CSF was positive, are similar to those seen in patients with smallpox encephalitis [13]. Symptoms of smallpox encephalitis include altered mental status, indifference, drowsiness, and semicomatose condition, whereas CSF findings rarely show biochemical abnormalities. Smallpox respiratory complications are common, reportedly related to inhalation as the virus´s transmission mechanism [15]. In the severe mpox cases presented here, we could prove lung involvement by imaging, PCR, and histopathology, even though mpox transmission mechanism was primarily by close contact.

As mentioned in the case descriptions, persistently positive PCR in blood was evidenced in cases 1, 2, and 3, with low ct (cycle threshold) values throughout all the disease course. Intense and continuous viremia has been pointed out as the potential mechanism for severe cases of variola major [13]. Moreover, persistent viremia seems to be the pathological cause of the progressive and disseminated course of disease in other Orthopoxvirus infections, as shown in studies on humans with diseases of cellular immunity [16–18]. Death, as hypothesized by Rao, also seems to occur secondary to severe viremia and toxemia as shown by elevated C-reative protein and persistent positive PCR in serum [13].

Lung and other organ involvement might reflect the widespread dissemination of the mpox virus in severe cases such as these. Cases shown in this report had a profound CD4^+^ T cell deficiency, which probably accounted for the severity of the disease. HIV-related immunosuppression appears to be a predisposing factor for mpox-related complications and death [5, 19]. In this series, the two patients with the most prolonged antiretroviral treatment duration survived, possibly indicating that immune reconstitution had already begun before mpox. The late-onset multiorgan dysfunction observed in our cases followed the sequence of events demonstrated by an aerosolized mpox model in monkeys [20].The reticuloendothelial system seems to be a critical factor in disseminating the virus, as shown by the early involvement of the spleen, lymph nodes, and tonsils. Also, lymphoid depletion, reduced number of NK cells, and overproduction of pro-inflammatory cytokines (cytokine storm) have been implicated in the immunopathological events of severe mpox [21]. Although these progression markers were not directly assessed, we observed profound lymphopenia and meager numbers of CD4^+^ cells as common factors in the cases presented here.

Histopathologic findings in lung biopsy highlight the extensive necrotic spectrum of disease seen in patients with disseminated disease. Although smallpox virus bronchopneumonia is usually referred to as suppurative pneumonia, distinct virus-associated changes also include necrosis of the alveolar epithelium [22]. This similarity with the smallpox virus raises the question of aerosolization as a potential transmission mechanism of mpox.

Immune reconstitution inflammatory syndrome (IRIS) is defined as a worsening of a condition after initiating antiretroviral therapy in HIV-infected patients [23]. Clinical and laboratory criteria have been proposed to aid in the diagnosis of IRIS [24]. In the present series of cases, IRIS was sustained based on CD4^+^ cell count < 200, the recent initiation of antiretroviral therapy, and rapid clinical deterioration. However, a decline in HIV viral load or an increase in CD4 cell count was not documented due to the short time between the start of antiretroviral therapy and death. Antiretroviral regimes used included integrase inhibitors, known for their fast decline in plasma HIV-RNA [25]. These patients may experience accelerated immune recovery, which might have contributed to a more severe inflammatory response [26]. In patients with mpox and previously untreated HIV infection, it is unknown when to initiate antiretrovirals.

The attempt to treat one of the patients in our series via passive immunization failed. Possible explanations for this include timing of plasma administration, lower than needed antibody titers (donor vaccinated, not convalescent), untreated concomitant HIV infection, and probable IRIS, among many others yet unknown. There is little clinical evidence for the current authorized therapies for mpox, such as tecovirimat, cidofovir, and brincidofovir [27]. A retrospective study reporting results of mpox infection treated with tecovirimat suggests faster symptom resolution for patients who received it earlier in the course of disease [28]. However, large randomized clinical trials evaluating antivirals, immunoglobulin, monoclonal antibodies, and other possible therapies are needed, especially for patients with severe disease.

In summary, we present the clinicopathological correlation of severely necrotic mpox, with assessment of viral presence and persistence in different body tissues of patients with HIV-infection. We encourage testing for undiagnosed HIV infection in patients presenting with mpox, and we support a comprehensive widespread vaccination in high risk-groups.

## Data Availability

Further patient data cannot be shared due to legal and ethical reasons.

## List of abbreviations

HIV: human immunodeficiency virus
PCR: polymerase chain reaction
bpm: beats per minute
rpm: respirations per minute
SpO2: oxygen saturation
ct: cycle threshold
CT: computed tomography
MRI: magnetic resonance imaging
IRB: investigation review board
CSF: cerebrospinal fluid
WBC: white blood cell
RBC: red blood cell
HSV: herpes simplex virus
PAS: periodic acid Schiff
RNA: ribonucleic acid

## References

[1] Sklenovska NVRM (2018). Emergence of Monkeypox as the most important Orthopoxvirus Infection in humans. Front Public Health.

[2] Thornhill JP, Barkati S, Walmsley S, Rockstroh J, Antinori A, Harrison LB, et al. Monkeypox Virus Infection in humans across 16 countries — April–June 2022. New England Journal of Medicine; 2022. 10.1056/NEJMoa2207323 35866746

[3] Thornhill JP, Palich R, Ghosn J, Walmsley S, Moschese D, Cortes CP (2022). Human monkeypox virus Infection in women and non-binary individuals during the 2022 outbreaks: a global case series. The Lancet.

[4] Mailhe M, Beaumont A-L, Thy M, Le Pluart D, Perrineau S, Houhou-Fidouh N et al. Clinical characteristics of ambulatory and hospitalized patients with monkeypox virus Infection: an observational cohort study. Clin Microbiol Infect. 2022. 10.1016/j.cmi.2022.08.012 PMC9533921 36028090

[5] Silva MST, Coutinho C, Torres TS, Peixoto E, Ismério R, Lessa F et al. Ambulatory and hospitalized patients with suspected and confirmed mpox: an observational cohort study from Brazil. Lancet Reg Health - Americas. 2022:100406. 10.1016/j.lana.2022.100406 PMC9904017 36776570

[6] Hoffmann C, Jessen H, Wyen C, Grunwald S, Noe S, Teichmann J et al. Clinical characteristics of monkeypox virus Infections among men with and without < scp > HIV: a large outbreak cohort in Germany. HIV Med. 2022. 10.1111/hiv.13378 36059149

[7] Català A, Clavo-Escribano P, Riera-Monroig J, Martín-Ezquerra G, Fernandez-Gonzalez P, Revelles-Peñas L (2022). Monkeypox outbreak in Spain: clinical and epidemiological findings in a prospective cross-sectional study of 185 cases*. Br J Dermatol.

[8] Mitjà O, Alemany A, Marks M, Lezama Mora JI, Rodríguez-Aldama JC, Torres Silva MS et al. Mpox in people with advanced HIV Infection: a global case series. The Lancet. 2023. 10.1016/S0140-6736(23)00273-8 36828001

[9] Caria J, Pinto R, Leal E, Almeida V, Cristóvão G, Gonçalves AC (2022). Clinical and epidemiological features of hospitalized and ambulatory patients with human monkeypox Infection: a retrospective observational study in Portugal. Infect Disease Rep.

[10] Menezes YR, Miranda ABD. Severe disseminated clinical presentation of monkeypox virus Infection in an immunosuppressed patient: first death report in Brazil. Volume 55. Revista da Sociedade Brasileira de Medicina Tropical. 2022. 10.1590/0037-8682-0392-2022 PMC9425919 36037315

[11] Alpalhão M, Sousa D, Frade JV, Patrocínio J, Garrido PM, Correia C et al. Human immunodeficiency virus Infection may be a contributing factor to monkeypox Infection: analysis of a 42-case series. J Am Acad Dermatol. 2022. 10.1016/j.jaad.2022.09.029 PMC9534227 36156305

[12] Riser AP, Hanley A, Cima M, Lewis L, Saadeh K, Alarcon J (2023). Epidemiologic and clinical features of Mpox-Associated deaths - United States, May 10, 2022-March 7, 2023. MMWR Morb Mortal Wkly Rep.

[13] Rao AR (1972). Smallpox. Bombay.

[14] Hazra A, Zucker J, Bell E, Flores J, Gordon L, Mitjà O et al. Mpox in people with past Infection or a complete vaccination course: a global case series. The Lancet Infectious Diseases. 10.1016/S1473-3099(23)00492-9 37678309

[15] Fenner F, Henderson DA, Arita I, Jezek Z, Ladnyi ID, World Health O. Smallpox and its eradication / F. Fenner ... [et al.]. Geneva: World Health Organization. 1988.

[16] Bray M, Buller M (2004). Looking back at Smallpox. Clin Infect Dis.

[17] Committee otAoFSNfLVV. Assessment of Future Scientific Needs for Live Variola Virus. 1993. 25101435

[18] Virus IoMCotAoFSNfLV. Clinical features of smallpox: National Academies Press (US); 1999 1999.

[19] Miller MJ, Cash-Goldwasser S, Marx GE, Schrodt CA (2022). Severe Monkeypox in Hospitalized patients - United States, August 10-October 10, 2022. MMWR Morb Mortal Wkly Rep.

[20] Tree JA, Hall G, Pearson G, Rayner E, Graham VA, Steeds K (2015). Sequence of pathogenic events in Cynomolgus macaques infected with Aerosolized Monkeypox Virus. J Virol.

[21] Li H, Huang QZ, Zhang H, Liu ZX, Chen XH, Ye LL (2023). The land-scape of immune response to monkeypox virus. EBioMedicine.

[22] Cann JA, Jahrling PB, Hensley LE, Wahl-Jensen V (2013). Comparative Pathology of Smallpox and Monkeypox in Man and macaques. J Comp Pathol.

[23] Bosamiya SS (2011). The immune reconstitution inflammatory syndrome. Indian J Dermatol.

[24] French MA, Price P, Stone SF (2004). Immune restoration Disease after antiretroviral therapy. Aids.

[25] Wijting IEA, Wit FWNM, Rokx C, Leyten EMS, Lowe SH, Brinkman K (2019). Immune reconstitution inflammatory syndrome in HIV infected late presenters starting integrase inhibitor containing antiretroviral therapy. EClinicalMedicine.

[26] Simon-Gozalbo A, Gamo-Guerrero M, Alonso-Garcia S, Mauleon-Fernandez C, Cuevas-Tascon G. Haemorrhagic monkeypox Infection in an immunosuppressed patient with human immunodeficiency virus: beyond the pustules. Clin Microbiol Infect. 2022. 10.1016/j.cmi.2022.09.017 PMC9534161 36206864

[27] Stafford A, Rimmer S, Gilchrist M, Sun K, Davies EP, Waddington CS (2023). Use of cidofovir in a patient with severe mpox and uncontrolled HIV Infection. Lancet Infect Dis.

[28] McLean J, Stoeckle K, Huang S, Berardi J, Gray B, Glesby MJ (2023). Tecovirimat Treatment of people with HIV during the 2022 Mpox Outbreak. Ann Intern Med.

